# Obituary—Dr. John P. Wilson

**Published:** 1868-03

**Authors:** 


### OBITUARY.

Died of hæmoptysis, on the 16th of March 1868, at the residence of Dr. W. H. Morgan, in Nashville, Tenn., Dr. John P. Wilson, in the 28th year of his age.

Dr. Wilson was a native of Pennsylvania, but for more than two years preceding his death, had resided in Nashville, and was a worthy member of the Nashville and the Tennessee Dental Associations. He was an ornament to the profession, and was no less distinguished for suavity of manners, and uniform gentlemanly deportment, than as a skillful practitioner of Dental Surgery.

